# Web 2.0 Applications in Medicine: Trends and Topics in the Literature

**DOI:** 10.2196/med20.3628

**Published:** 2015-04-01

**Authors:** Christophe Boudry

**Affiliations:** ^1^ Media Normandie Normandy University, University of Caen Basse-Normandie Caen Cedex France; ^2^ Unité Régionale de Formation à l’Information Scientifique et Technique de Paris Ecole nationale des Chartes Paris France; ^3^ Laboratory Dispositifs d’Information et de Communication à l’Ère Numérique Conservatoire National des Arts et Métiers Paris France

**Keywords:** Social media, Internet, Health information management, bibliometrics, Medline, Blogging, Medical Informatics

## Abstract

**Background:**

The World Wide Web has changed research habits, and these changes were further expanded when “Web 2.0” became popular in 2005. Bibliometrics is a helpful tool used for describing patterns of publication, for interpreting progression over time, and the geographical distribution of research in a given field. Few studies employing bibliometrics, however, have been carried out on the correlative nature of scientific literature and Web 2.0.

**Objective:**

The aim of this bibliometric analysis was to provide an overview of Web 2.0 implications in the biomedical literature. The objectives were to assess the growth rate of literature, key journals, authors, and country contributions, and to evaluate whether the various Web 2.0 applications were expressed within this biomedical literature, and if so, how.

**Methods:**

A specific query with keywords chosen to be representative of Web 2.0 applications was built for the PubMed database. Articles related to Web 2.0 were downloaded in Extensible Markup Language (XML) and were processed through developed hypertext preprocessor (PHP) scripts, then imported to Microsoft Excel 2010 for data processing.

**Results:**

A total of 1347 articles were included in this study. The number of articles related to Web 2.0 has been increasing from 2002 to 2012 (average annual growth rate was 106.3% with a maximum of 333% in 2005). The United States was by far the predominant country for authors, with 514 articles (54.0%; 514/952). The second and third most productive countries were the United Kingdom and Australia, with 87 (9.1%; 87/952) and 44 articles (4.6%; 44/952), respectively. Distribution of number of articles per author showed that the core population of researchers working on Web 2.0 in the medical field could be estimated at approximately 75. In total, 614 journals were identified during this analysis. Using Bradford’s law, 27 core journals were identified, among which three (Studies in Health Technology and Informatics, Journal of Medical Internet Research, and Nucleic Acids Research) produced more than 35 articles related to Web 2.0 over the period studied. A total of 274 words in the field of Web 2.0 were found after manual sorting of the 15,878 words appearing in title and abstract fields for articles. Word frequency analysis reveals “blog” as the most recurrent, followed by “wiki”, “Web 2.0”, ”social media”, “Facebook”, “social networks”, “blogger”, “cloud computing”, “Twitter”, and “blogging”. All categories of Web 2.0 applications were found, indicating the successful integration of Web 2.0 into the biomedical field.

**Conclusions:**

This study shows that the biomedical community is engaged in the use of Web 2.0 and confirms its high level of interest in these tools. Therefore, changes in the ways researchers use information seem to be far from over.

## Introduction

Over the past two decades, the World Wide Web has changed researchers’ habits. These changes were further expanded when “Web 2.0” became popular in 2005 [1], providing tools and platforms that facilitate user collaboration, user-generated content, and data sharing. These tools have gradually influenced the world of research [2,3], especially in biology and medicine [4-9], and their use is increasingly common, notably with the arrival of “digital natives” in laboratories [10,11].

Bibliometrics is a helpful and widely used tool for describing patterns of publication and interpreting temporal evolutions and the geographical distribution of research in a given field. However, few studies employing bibliometrics have been carried out on the correlative nature of scientific literature and Web 2.0. A bibliometric analysis was performed in 2009 by Chu and Xu [12] on a set of 1718 documents relating to Web 2.0 using several databases. It was found that Web 2.0 is a rapidly developing area, with medicine and sociology being the major contributing disciplines to the scholarly publications. In 2011, Aharony [13] performed a statistical descriptive analysis and a thorough content analysis of descriptors and journal titles in the field of library and information science in a study of 472 articles. They focused on the subject of Web 2.0 and its main applications. Main findings revealed that the percentage of articles related to Web 2.0 was low, and showed a close link between Web 2.0 and library topics. In the field of medicine, Van De Belt et al [6] performed a systematic literature review in 2010 of electronic databases (PubMed, Scopus, CINAHL) and gray literature on the Internet using search engines to identify unique definitions of Health 2.0/Medicine 2.0 and recurrent topics within the definitions. The analysis was done on 1937 documents and they concluded that Health 2.0/Medicine 2.0 were still developing areas, and that there was still no general consensus regarding the definition of Health 2.0/Medicine 2.0.

The aim of the present study was to provide an overview of Web 2.0 implications in the biomedical literature and to answer the following questions: What is the growth rate of biomedical literature on Web 2.0?; What are the key publications, countries, and authors in the field?; Which Web 2.0 terms are the most recurrent in biomedical literature?; and, Are the various applications of Web 2.0 expressed in the biomedical literature? Established bibliometric methods have been used to perform the present study. One example is the identification of core journals using Bradford’s law of scattering which has, to the best of our knowledge, never been done to study literature related to Web 2.0.

## Methods

The search for papers to be included in this study was carried out on February 7, 2013, using the PubMed database [14], developed by the National Center for Biotechnology Information (NCBI) at the National Library of Medicine (NLM). Keywords used in the search were chosen since they were known to be representative of Web 2.0 applications [7,12,13,15]. Search strategy was built around identifying keywords in medical subject headings (MeSH) and completed, in the absence of results, by a text search in the title and abstract fields. When necessary, keywords were accompanied by a truncation to bring in all possible variants. The study was limited to original research articles corresponding to “Journal articles” shown under the “Publication type” field.

The final search strategy was the following: (“social networking”[MeSH Terms] AND (web [Title/Abstract] OR internet [Title/Abstract])) OR (“web 2.0” [Title/abstract] OR “Medicine 2.0” [Title/abstract] OR “Health 2.0” [Title/abstract] OR “Biology 2.0” [Title/abstract] OR “science 2.0” [Title/abstract] OR Social Media [MH] OR Syndication [Title/Abstract] OR wiki [Title/Abstract] OR Blogging [MeSH Terms] OR blog* [Title/Abstract] OR microblogg* [Title/Abstract] OR Cloud computing [Title/Abstract] OR folksonom* [Title/Abstract] OR social bookmark* [Title/Abstract]) AND (1951:2012 [DP]) AND (journal article [PT]), where MeSH stands for “Medical Subject Headings”, DP “Date of Publication”, and PT “Publication Type”.

Data downloaded from PubMed in Extensible Markup Language (XML) were processed through developed hypertext preprocessor language (PHP) scripts, then were imported to Microsoft Excel 2010. All articles were manually reviewed by the author of this article and those not related to Web 2.0 were eliminated. When no abstract was available for a reference, PubMed “Related citations” were consulted to determine the eligibility of the article in the present study.

Microsoft Excel served for assessing the growth of literature, for journals, language of publication, authorship pattern, and number of publications per country. The average yearly growth rate was calculated as the mean percentage of annual growth for the period studied, with average yearly growth rate=(Current year total - Previous year total)/Previous year total [16,17].

Average yearly growth rate and percentage of articles published in English were also calculated for the whole PubMed database for the period 2002-2012. This period was chosen because it corresponds to the period where articles related to Web 2.0 were found in this study.

Bradford’s law of scattering has been used extensively in the information science literature to describe the dispersion of articles in any scientific field [18] and to identify core journals of serial titles [16,19,20]. Bradford’s law states that “if scientific journals are arranged in order of decreasing productivity of articles on a given subject, they may be divided into a nucleus of periodicals more particularly devoted to the subject and several groups or zones containing the same number of articles as the nucleus, when the numbers of periodicals in the nucleus and succeeding zones will be as 1: n : n^2^” [21]. This means that “Bradford’s law predicts that the number of journals in the second and third zones will be n and n^2^ times larger than the first zone respectively, and therefore, it should be possible to predict the total number of journals containing articles on a subject once the number in the core and middle zone of journals is known” [22]. To identify the core journals and predict the number of journals containing articles related to Web 2.0, we applied Bradford’s law by dividing the publication frequency ranked journals into three groups, with each group containing approximately the same number of articles.

The Journal Citation Reports (Thomson Reuters) was used for Impact Factor determination. For the determination of affiliation of authors, England, Scotland, Northern Ireland, and Wales were clustered into the United Kingdom. Words from both title and abstract fields were recovered for keyword frequency calculation using TextSTAT 2.9 software [23]. Words or expressions were manually sorted to extract those relating to Web 2.0. Similar words—differing by the singular/plural, upper/lower case—were aggregated (eg, wiki/wikis, facebook/Facebook). Words thus obtained were manually sorted into eight categories: one general category and seven others corresponding to blog, cloud computing, microblogging, social bookmarking/document sharing, social network, syndication, and wiki.

## Results

### Overview

The publication search turned in a total of 1578 references. After manual sorting and elimination of inappropriate references, 1347 articles were retained for inclusion in the study.

### Growth of Literature

**Figure 1:**
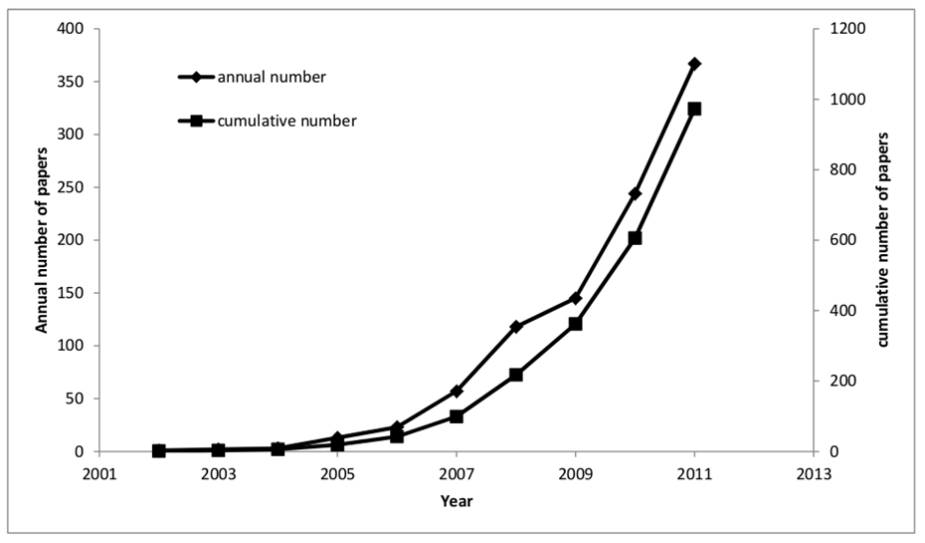
Growth of literature (annual number and cumulative number).

As shown in Figure 1, Web 2.0 references, starting in 2002 with one article, had risen to 1000 per year by 2009 and continued to grow throughout 2011 (2012, being incomplete, is not represented). The average annual growth rate for the period 2002-2011 was 106.30% for Web 2.0 related articles, and 6.27% for the whole PubMed database for the same period.

### Journals

**Figure 2:**
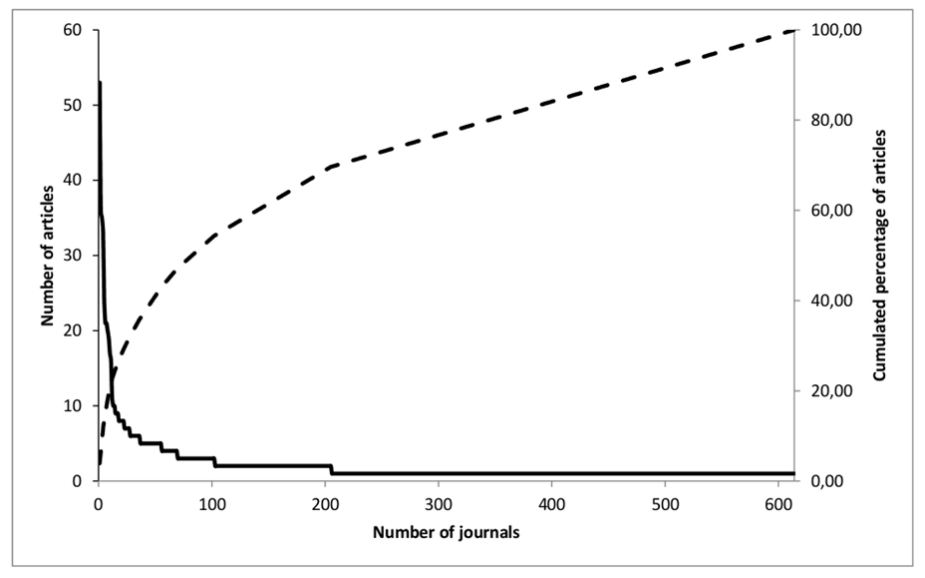
Distribution of number of articles per journal (solid line) and cumulated percentage of articles (dotted line).

A total of 614 journals were identified during this analysis. As shown in Figure 2 and Table 1, one-third of the published articles were found in a mere 27 journals (27/614; 4.4%). This first third represents the journals that published the most articles (between 7 and 53 articles on the period studied) and that are presumed to be of highest interest for researchers interested in Web 2.0 (“core journals”). The middle third corresponds to the journals (178/614; 29.0% of journals) that published an average amount of articles, and the last third includes the “long tail” of journals (409/614; 66.6% of journals) that published one article and must be regarded as being of least importance. The theoretical ratio of number of journals (43.4) and the theoretical number of journals in the last third (1172) were higher than the values obtained experimentally (15.1 and 409, respectively).

Table 2 presents the 38 journals that have published more than six articles and their Impact Factor (IF) when available.

**Table 1:** Bradford zones of scattering for Web 2.0 literature.

Zones	Number of journals	Percentage of journals	Number of articles	Cumulative number of articles (%)	Description	Ratio (number of journals)	Theoretical ratio (1:n:n^2^)	Theoretical number of journals
Core journals	27	4.4%	428	428 (31.7)	Producing <53 and ≥7 articles	1	1	27
Middle	178	29.0%	510	938 (61.7)	Producing ≤6 and ≥2 articles	n=6.6	n=6.6	178
Last	409	66.6%	409	1347 (100)	Producing 1 article	n^2^=15.1	n^2^=43.4	1172
Total	614	100.0%	1347					1377

**Table 2:** Major Web 2.0 publishing journals (journals publishing more than six articles).

Journal	Articles, n=1347 n (%)		Impact factor			MeSH terms^b^
Studies in Health Technology and Informatics^a^	53 (3.93)		N/A			Biomedical Technology; Medical Informatics
Journal of Medical Internet Research^a^	36 (2.67)		3.768			Information Services; Internet; Medical Informatics; Research
Nucleic Acids Research^a^	35 (2.60)		8.278			Nucleic Acids
Cyberpsychology, Behavior and Social Networking^a^	33 (2.45)		N/A			Behavior; Computer Communication Networks/utilization; Multimedia/utilization; Psychology, Social; User-Computer Interface
AMIA. Annual Symposium proceedings / AMIA Symposium. AMIA Symposium^a^	24 (1.78)		N/A			Medical Informatics Applications; Medical Informatics Computing
BMC Bioinformatics^a^	21 (1.56)		3.024			Computational Biology
Medical Reference Services Quarterly^a^	21 (1.56)		N/A			Information Services; Information Systems; Libraries, Medical; Library Services
Medical Teacher^a^	20 (1.48)		1.824			Education, Medical
Nurse Educator^a^	19 (1.41)		0.562			Education, Nursing
PLoS One^a^	17 (1.26)		3.73			Medicine; Science
Bioinformatics (Oxford, England)^a^	16 (1.19)		5.323			Computational Biology; Genome
Health Information and Libraries Journal^a^	11 (0.82)		N/A			Libraries, Medical; Medical Informatics
Genome Biology^a^	10 (0.74)		10.288			Biology; Genetics; Genome
Journal of Medical Systems^a^	10 (0.74)		1.783			Computers; Delivery of Health Care; Information Systems
BMJ (Clinical research ed.)^a^	9 (0.67)		17.215			Medicine
Journal of Digital Imaging^a^	9 (0.67)		1.1			Computer Systems ; Radiographic Image Enhancement; Radiology Information Systems
Journal of Health Communication^a^	9 (0.67)		N/A			Communication; Health Education; Health Promotion; Health Services; Health
Cyberpsychology & Behavior: the impact of the Internet, multimedia and virtual reality on behavior and society^a^	8 (0.59)		N/A			Behavior; Computer Communication Networks/utilization; Multimedia/utilization; Psychology, Social; User-Computer Interface
Health Communication^a^	8 (0.59)		N/A			Communication; Health
Nature^a^	8 (0.59)		38.597			Science
The Journal of Medical Practice Management : MPM^a^	8 (0.59)		N/A			Practice Management, Medical
Vaccine^a^	8 (0.59)		3.492			Vaccines
American Journal of Pharmaceutical Education^a^	7 (0.52)		N/A			Education; Pharmacy
Annual International Conference of the IEEE Engineering in Medicine and Biology Society^a^	7 (0.52)		N/A			Biomedical Engineering
Journal of Dental Education^a^	7 (0.52)		0.989			Education, Dental
Journal of the Medical Library Association : JMLA^a^	7 (0.52)		N/A			Information Services; Libraries, Medical; Library Science
Medical Education^a^	7 (0.52)		3.546			Education, Medical
Caring: National Association for Home Care magazine	6 (0.45)		N/A			Health Services for the Aged; Home Care Services; Long-Term Care
Database: the journal of biological databases and curation	6 (0.45)		4.2			Computational Biology
Journal of the American Medical Informatics Association : JAMIA	6 (0.45)		3.571			Medical Informatics Applications; Medical Informatics
Nursing education perspectives	6 (0.45)		N/A			Education, Nursing; Nursing
PLoS computational biology	6 (0.45)		4.867			Computational Biology
Science (New York, N.Y.)	6 (0.45)		31.027			Science
The Journal of Adolescent Health: Official Publication of the Society for Adolescent Medicine	6 (0.45)		2.966			Adolescent Medicine
The Journal of Nursing Education	6 (0.45)		1.133			Education, Nursing
Tobacco Control	6 (0.45)		4.111			Smoking/prevention & control; Tobacco Use; Disorder/prevention & control; Tobacco

^a^Core journals according to Bradford’s law of scattering.

^b^MeSH terms used in the catalog of the National Library of Medicine to describe the journal.

### Language of Publication

A total of 1355 declared languages were retrieved among the 1347 articles. This disparity could be explained by the fact that some articles have two languages declared in the language field in PubMed. The most commonly used language was English (1301/1355; 96.01%), followed by French (17/1355; 1.25%); Spanish (12/1355; 0.89%); German (8/1355; 0.59%); Italian (4/1355; 0.30%); Dutch (3/1355; 0.22%); Japanese, Portuguese (2/1355; 0.15%); and Danish, Greek, Hungarian, Norwegian, Polish, and Swedish (1/1355; 0.07%). The percentage of publications in English for the whole PubMed database was 90.84% for this given period (2002-2011).

### Geographical Repartition of Authors (Country Contributions)

For 395 of the 1347 articles (29.32%), it was impossible to identify the contributing country because the author claimed no affiliation and the articles failed to name the country of publication. Therefore, only 952 of the articles studied could be linked to countries. Table 3 shows the number of papers published per country.

The United States was by far the predominant country for authors, with 514 articles (514/952; 54.0%). The second most productive country was the United Kingdom with 87 articles (87/952; 9.1%). Authors from Europe produced 264 articles (264/952; 27.7 %).

**Table 3:** Number and percentage of articles published per country relative to the affiliation of authors (n=952).

Country	Articles, n (%)
United States	514 (54.0)
United Kingdom	87 (9.1)
Australia	44 (4.6)
Canada	41 (4.3)
China	40 (4.2)
Germany	34 (3.6)
Spain	26 (2.7)
Netherlands	21 (2.2)
France	14 (1.5)
Italy	14 (1.5)
Greece	11 (1.2)
Japan	11 (1.2)
New Zealand	10 (1.1)
Switzerland	10 (1.1)
India	9 (0.9)
Israel	9 (0.9)
Norway	8 (0.8)
Sweden	8 (0.8)
Portugal	6 (0.6)
Belgium	4 (0.4)
Ireland	4 (0.4)
Austria	3 (0.3)
Brazil	3 (0.3)
Bulgaria	3 (0.3)
Egypt	3 (0.3)
Romania	3 (0.3)
Turkey	3 (0.3)
Luxembourg	2 (0.2)
South Africa	2 (0.2)
Argentina	1 (0.1)
Czech Republic	1 (0.1)
Poland	1 (0.1)
Singapore	1 (0.1)
Slovakia	1 (0.1)

### Number of Papers Per Author

In total, 4209 authors were found for the 1347 articles retained, corresponding to 3762 different authors. The great majority of authors (91.54%; 3444/3762) wrote only one article, 6.51% (245/3762) wrote two articles, whereas 73 (1.94%; 73/3762) wrote three or more. The maximum number of articles written by one author was 14.

### Word Frequency Analysis

A total of 274 words in the field of Web 2.0 were found after manual sorting of the 15,878 words belonging to title and abstract fields. Similar words differing by singular/plural, upper/lower case were aggregated and 99 words finally obtained. As shown in Tables 4 and 5, word frequency analysis reveals that the ten most frequent words or expressions are “blog” followed by “wiki”, “Web 2.0”,”social media”, “Facebook”, “social networks”, “blogger”, “cloud computing”, “Twitter”, and “blogging”.

In the general category, “Web 2.0” was the most common expression followed by e-health, Health 2.0, and Medicine 2.0. “Blog” was the predominant category of any Web 2.0 application encountered in the biomedical literature, followed by social networks and wiki (1279, 1199, and 803 occurrences, respectively). Micro-blogging, cloud computing, social bookmarking/document sharing, and syndication were much less represented with 332, 260, 183, and 175 occurrences, respectively.

**Table 4:** Word frequency for general, blog, social network, and wiki categories.

General		Blog		Social network		Wiki	
Word	n	Word	n	Word	n	Word	n
Web 2.0	542	Blog	800	social media	472	Wiki	536
e-health	71	Blogger	259	Facebook	336	Wikipedia	108
Health 2.0	39	Blogging	181	Social network	327	Crowdsourcing	21
Medicine 2.0	36	Blogosphere	30	MySpace	29	MediaWiki	18
Science 2.0	8	Nonbloggers, Blogroll	2	Second life	21	SubtiWiki	17
e-Research	3	Medbloggers, blogspot, Bloglines, blogorrhea, blogsearch	1	LinkedIn	9	WikiPathways	10
O’Reilly	2			PatientsLikeMe	5	myExperiment	9
eScience	1					ArrayWiki	8
						EcoliWiki, SNPedia	7
						Pathowiki, PDBWiki	6
						Channelpedia, UMMedWiki	5
						WikiGenes, WikiPharma, Bowiki, TWiki, Proteopedia,	4
						Casepedia, SEQwiki, Gene_Wiki, WikiBuild	3
						WikiProteins, OperonWiki, meta-wiki, OpenToxipedia, CHDWiki, Wikisource, Wikibooks, WikiOpener, RAASWiki	2
						Genewikiplus, WikiMedia, wikispaces, Clinfowiki, wikiprofessional, OpenWetWare, gowiki, sbwWiki, WikiTrust, Wikipedians, Medi-wiki	1
Total	702	Total	1279	Total	1199	Total	803

**Table 5:** Word frequency for microblogging, cloud computing, social bookmarking/document sharing, and syndication categories.

Micro blogging		Cloud computing		Social Bookmarking/ document sharing		Syndication	
Word	n	Word	n	Word	n	Word	n
Twitter	205	cloud computing	209	YouTube	89	podcast	69
Tweet	73	Amazon	33	Tag	31	RSS	56
micro-blogging	31	CloudLCA	6	social bookmarking	29	syndication	37
Weibo	12	CloudMan	3	Tagging	13	podcasting	11
Tweeting	6	SurveyMonkey, Netvibes, CloudBioLinux, GeoCommons	2	Folksonomy	11	uBioRSS	2
iScience	3	CloudBurst	1	video-sharing	4		
micro-blog	2			Delicious	3		
				Digg, CiteULike, Slideshare	1		
Total	332	Total	260	Total	183	Total	175

## Discussion

### Principal Findings

The appearance of literature relating to Web 2.0 in the biomedical field is recent, and correlates with the year 2005, when Web 2.0 became popular [1]. Some Web 2.0 applications existed before this date, which is why some articles were identified earlier. The scientific production of Web 2.0 really started in 2006 and has been growing rapidly ever since. The comparison of average annual growth rate for Web 2.0 related articles and for the whole PubMed database (106.30% and 6.27%, respectively) has confirmed that the topic continues to be of much interest to the biomedical community.

Using Bradford’s law of scattering, the theoretical ratio of number of journals (43.4) and theoretical number of journals in the last third (1172) were higher than the values obtained experimentally (15.1 and 409, respectively). Thus, articles related to Web 2.0 are published in a lesser number of journals (n=614) than the expected Bradford theoretical value (n=1377). This can be explained by the innovative nature of the subject studied, which has not yet been taken into account by a great number of journals.

In the list of the 38 journals that published more than six articles, including core journals according to Bradford’s law (Table 2), widely disseminated journals with high impact factors (IF) are present: three are in the 100 journals that have the highest IF (Nature, Science, BMJ), and one is in the top 10 (Nature, IF=38.597). Six journals out of 38 (16%) have an IF greater than 5, whereas, in the complete Journal Citation Reports, the percentage with an IF higher than 5 is 6.2%, indicating that journals with significant scientific influence are interested in Web 2.0. Only one of the 38 journals that published more than six articles, the Journal of Medical Internet Research, specializes in Internet studies. It should be noted that many journals with educational objectives are in this list, because Web 2.0 tools and techniques are new and their comprehension and utilization require a learning period.

English was by far the most predominant language of the articles included in the study, and the percentage of articles in English was higher compared to the entire PubMed database (96.01% and 90.84%, respectively). This can be explained by the fact that English is the official language for scientific publications in most countries. As mentioned elsewhere [24,25], PubMed is a US database, so it may have introduced a bias because most of the journals indexed are written in English, which could accentuate its predominance. These observations match those of other studies done with bibliometrics in fields where information is predominantly found in English.

The United States was by far the most productive country. Europe came second globally, whereas Africa and South America were very poorly represented.

Distribution of number of articles per author shows that the great majority of authors (91.54%; 3444/3762) wrote only one article, whereas 73 (1.94%; 73/3762) wrote three or more. Thus, the core population of researchers working on Web 2.0 in the biomedical field can be estimated to approximately 75.

Considering generalist terms or expressions, the word frequency analysis reveals “Web 2.0” as the most common term, followed by “e-health”, “Health 2.0”, and “Medicine 2.0”, which are the expressions most commonly used to describe Web 2.0 technologies applied in this field [8].

The most represented category of the eight was blog (1279 occurrences). This can be explained by the fact that blogs are among the oldest Web 2.0 applications and the facility of their implementation has established their popularity. Quite logically, in the second category, “social network”, the well-known Facebook was by far the most represented. Among wikis, Wikipedia was the most represented term. The high number of terms in this category is due to the many applications based on wiki platforms developed by researchers, and most of the articles related to these terms are actually presentations of these applications. The most cited micro-blogging application was, as expected, Twitter, confirming its high popularity. Cloud computing applications, currently on the rise, are also well represented, even though access to them is fairly recent compared to that of blogs or wikis. Amazon, best known for its online shopping website, is cited because it also offers solutions for the development of cloud computing applications. The category social bookmarking / document sharing was predominantly represented by YouTube. Unexpectedly, social bookmarking sites specially developed for the scientific field were scarce (eg, Citeulike), or simply not present (eg, Connotea or Bibsonomy). The same can be said of other categories in which the most represented terms were related to popular applications (Facebook, YouTube, Wikipedia, and Twitter, respectively the first, third, sixth, and tenth most consulted sites in the world according to [26]). Of note, apart from wikis, applications specifically developed for science, biology, or medicine were rare (eg, PatientsLikeMe) or not represented in every category (eg, researchblogging.org for blogs, Researchgate and Academia for social networks).

### Limitations

One should be aware that this study presents some limits: for even if PubMed is widely used for bibliometric analysis, it does not contain all biomedical journals [24], and some relevant articles may have been omitted. Furthermore, the methodology for identifying the country of authors (PubMed) indicates only one country per article and fails to identify transnational research. Moreover, some articles (395/1347; 29.32%) did not mention any country of affiliation for authors. Therefore, the geographical repartition of the latter might be underestimated in some locations [27]. Furthermore, some Web 2.0 applications, specifically developed for biology or medicine, may not have been retrieved by the search because they were only named and not described as Web 2.0 applications in articles.

### Conclusions

This paper presents an exploration of the geographical distribution and temporal trends of the biomedical literature related to Web 2.0 found in PubMed, together with an analysis of related words and expressions. The study indicates the ongoing expansion of a field currently dominated by the United States. All categories of Web 2.0 applications abound within the literature, indicating that Web 2.0 has been integrated into the biomedical field. Of note, applications developed specifically for biology and medicine were less represented than their generalist counterparts (eg, Facebook, Twitter). The study of articles published clearly shows a great diversity of journals, including those with significant scientific influence, displaying interest in Web 2.0, and confirms the high level of interest the topic holds for the biomedical community. Therefore, the changes in the informational uses of researchers, initiated by the arrival of the World Wide Web and continued by Web 2.0, seem to be far from over.

## Abbreviations

IF: impact factor
MeSH: medical subject headings
PHP: hypertext preprocessor language
XML: Extensible Markup Language

## References

[1] O’Reilly T (2005). What Is Web 2.0?.

[2] Shneiderman B (2008). Computer science. Science 2.0. Science.

[3] Whitmire A (2013). Thoughts on “eResearch”: a Scientist’s Perspective. JESLIB.

[4] Giustini D (2006). How Web 2.0 is changing medicine. BMJ.

[5] Boudry C (2012). ["Biology/medicine 2.0” : an overview]. Med Sci (Paris).

[6] Van De Belt TH, Engelen LJ, Berben SA, Schoonhoven L (2010). Definition of Health 2.0 and Medicine 2.0: a systematic review. J Med Internet Res.

[7] Eysenbach G (2008). Medicine 2.0: social networking, collaboration, participation, apomediation, and openness. J Med Internet Res.

[8] Oh H, Rizo C, Enkin M, Jadad A (2005). What is eHealth?: a systematic review of published definitions. World Hosp Health Serv.

[9] Gardois P, Colombi N, Grillo G, Villanacci MC (2012). Implementation of Web 2.0 services in academic, medical and research libraries: a scoping review. Health Info Libr J.

[10] Collins E, Hide B (2010). Use and relevance of web 2.0 resources for researchers. Publishing in the networked world: Transforming the Nature of Communication.

[11] Procter R, Williams R, Stewart J, Poschen M, Snee H, Voss A (2010). Adoption and use of Web 2.0 in scholarly communications. Philos Trans R Soc A-Math Phys Eng Sci.

[12] Chu H, Xu C (2009). Web 2.0 and its dimensions in the scholarly world. Scientometrics.

[13] Aharony N (2011). Web 2.0 in the professional LIS literature: An exploratory analysis. J of Librariansh and Inf Sci.

[14] (2013). Home - PubMed - NCBI.

[15] Hughes B, Joshi I, Wareham J (2008). Health 2.0 and Medicine 2.0: tensions and controversies in the field. J Med Internet Res.

[16] Deshazo JP, Lavallie DL, Wolf FM (2009). Publication trends in the medical informatics literature: 20 years of “Medical Informatics” in MeSH. BMC Med Inform Decis Mak.

[17] Krishnamoorthy G, Ramakrishnan J, Devi S (2009). Bibliometric analysis of literature on diabetes (1995-2004). Annals of Library and Information Studies.

[18] Goffman W, Warren KS (1969). Dispersion of papers among journals based on a mathematical analysis of two diverse medical literatures. Nature.

[19] Goffman W, Morris TG (1970). Bradford's law and library acquisitions. Nature.

[20] Tsay M, Yang Y (2005). Bibliometric analysis of the literature of randomized controlled trials. J Med Libr Assoc.

[21] Bradford S (1948). Documentation.

[22] Nash-Stewart CE, Kruesi LM, Del Mar CB (2012). Does Bradford's Law of Scattering predict the size of the literature in Cochrane Reviews?. J Med Libr Assoc.

[23] (2013). TextSTAT :: Niederländische Philologie FU Berlin.

[24] Uthman OA (2010). Pattern and determinants of HIV research productivity in sub-Saharan Africa: bibliometric analysis of 1981 to 2009 PubMed papers. BMC Infect Dis.

[25] Ugolini D, Neri M, Casilli C, Ceppi M, Canessa PA, Ivaldi GP, Paganuzzi M, Bonassi S (2010). A bibliometric analysis of scientific production in mesothelioma research. Lung Cancer.

[26] (1996). Alexa Top 500 Global Sites.

[27] Della Mea V (2011). 25 years of telepathology research: a bibliometric analysis. Diagn Pathol.

